# Expanding the case for gender-neutral human papillomavirus vaccination in South Africa: Emerging neonatal and ethical considerations

**DOI:** 10.4102/phcfm.v17i1.5035

**Published:** 2025-09-30

**Authors:** Vanessa C. Scheepers, Jillian Gardner

**Affiliations:** 1 School of Clinical Medicine, Faculty of Health Sciences, University of the Witwatersrand, Johannesburg, South Africa; 2 Department of Nursing and Midwifery, Faculty of Medicine and Health Sciences, Stellenbosch University, Bellville, South Africa

**Keywords:** human papillomavirus, neonatal outcomes, maternal transmission, paternal transmission, gender-neutral vaccination, ethical concerns

## Abstract

Human papillomavirus (HPV) is widely recognised for its role in causing cervical cancer, prompting many countries, including South Africa, to prioritise girls in school-based vaccination programmes. This short report presents an exploratory case for expanding HPV vaccination to adolescent boys as well, drawing on emerging, but still limited evidence of maternal and paternal HPV transmission to neonates. Although current data on neonatal risks are preliminary, the possibility of vertical transmission and associations with respiratory papillomatosis, preterm birth and fertility issues warrant further investigation. Beyond neonatal outcomes, gender-neutral HPV vaccination offers ethical and public health benefits by promoting equitable protection, enhancing herd immunity and addressing transmission dynamics. Recent advances, including the World Health Organization-endorsed single-dose schedules and the availability of affordable vaccines, provide opportunities to revisit cost-effectiveness analyses. We recommend further context-specific research and modelling to quantify the long-term benefits of gender-neutral strategies in South Africa and similar settings.

## Introduction

Human papillomavirus (HPV) is widely recognised for its role in causing cervical cancer, prompting countries such as South Africa to offer free HPV vaccination to school-going girls aged 9 to 14 years.^1^ This approach aligns with the World Health Organization’s (WHO) recommendation to prioritise girls in efforts to reduce the global burden of cervical cancer.^2^ However, emerging evidence shows that the effects of HPV extend beyond cervical pathology. The role of HPV in pregnancy-related complications and potential transmission to neonates has gained growing attention.^3,4,5,6^ Although girls-only HPV vaccination strategies may be justified by feasibility and cost-effectiveness,^1,2^ they overlook the contribution of male partners to HPV transmission and the associated risks of vertical transmission.^3,6,7^ While the current evidence on neonatal outcomes is still limited in scope and strength, it highlights critical gaps in our understanding of parent-to-child transmission. A gender-neutral vaccination strategy that includes adolescent boys may yield significant long-term benefits, including the prevention of HPV-related complications in neonates and across the population.^8^ This short report presents an exploratory public health and ethical case for expanding HPV vaccination to include boys in South Africa.

## Background on human papillomavirus and vertical transmission

Human papillomavirus is the most prevalent sexually transmitted infection globally, affecting sexually active men and women.^2^ Of the more than 200 identified HPV types, approximately 40 cause genital infections.^9^ These types are broadly categorised into high-risk strains, such as HPV 16 and 18, which are strongly linked to cancers of the cervix, anus, penis and oropharynx. Low-risk strains, such as HPV 6 and 11, are associated with genital warts.^9^ While sexual contact remains the primary mode of transmission, non-sexual routes, including vertical transmission from mother to child during pregnancy, delivery and early postpartum contact, are increasingly acknowledged.^3^

Studies have detected HPV deoxyribonucleic acid (DNA) in amniotic fluid, placental tissues, umbilical cord blood and the respiratory tract of neonates.^3,5^ Although vertical transmission does not always lead to active disease, its association with conditions such as recurrent respiratory papillomatosis (RRP) and potential links to miscarriage, premature rupture of membranes and preterm birth warrants further study.^3,4,5^ Notably, both low-risk (e.g. HPV 6 and 11) and high-risk (e.g. HPV 16 and 18) strains are implicated in these adverse outcomes.^3^ However, robust population-level data quantifying the extent of these effects, especially in South Africa, are still lacking.

## Maternal and paternal roles in human papillomavirus transmission

The role of maternal HPV infection in neonatal outcomes is well studied.^3,4,5^ However, research has highlighted the potential significance of paternal infection.^3,6,7^ Men can carry and transmit HPV asymptomatically, and paternal infection has been associated with poor semen quality, reduced fertility, recurrent pregnancy loss and possibly the transmission of high-risk HPV strains via spermatozoa.^6,7^

This dual parental contribution suggests that girls-only HPV vaccination strategies are insufficient to entirely disrupt the transmission cycle. If both parents can transmit HPV, sexually or vertically, then preventative measures must address both sexes. Vaccinating adolescent boys can significantly reduce the overall burden of HPV in the population,^8,10^ thus lowering the risk of horizontal (person-to-person) and vertical (mother-to-child) transmission. Nonetheless, further research is necessary to clarify the causal pathways and quantify the impact of paternal HPV infection.

## Challenges associated with girls-only human papillomavirus vaccination strategies

The WHO’s recommendation to prioritise girls in HPV vaccination programmes is based on epidemiological data and resource considerations.^2^ According to the WHO, girls-only vaccination is effective in lowering cervical cancer incidence, particularly in low- and middle-income countries (LMICs).^2^ However, this approach overlooks the role of males in maintaining HPV transmission and fails to offer direct protection to boys and their future offspring.

Girls-only HPV vaccination strategies rely on the assumption of high vaccine uptake among girls and limited HPV exposure from unvaccinated males,^1,2^ conditions that are often challenging to achieve in real-world settings. Herd immunity is only achieved when a sufficient proportion of a population is vaccinated, effectively limiting virus transmission.^8^ Therefore, girls-only programmes may fall short of achieving herd immunity and perpetuate HPV circulation among unvaccinated groups. The exclusion of boys creates significant gaps in prevention, specifically in preventing non-cervical HPV-related diseases and potential transmission during conception, pregnancy and birth.

## Public health and ethical justifications for gender-neutral vaccination

From a public health perspective, gender-neutral HPV vaccination increases herd immunity and reduces the overall prevalence of HPV within the population.^8,10^ Modelling studies indicate that gender-neutral vaccination is more effective in preventing HPV-related diseases than girls-only strategies, especially in settings where vaccine uptake among girls is suboptimal.^8^

While it is acknowledged that cervical cancer disproportionately affects women, gender-neutral vaccination promotes ethical fairness by reducing preventable disease burdens across genders and interrupting transmission to future generations.^11,12,13,14^ Gender-neutral HPV vaccination strategies also align with utilitarian and deontological frameworks by striving to minimise harm and uphold moral obligations to protect children from preventable infections.^11,12,13,14^

Economically, the upfront cost of expanding vaccination coverage may be balanced by long-term healthcare savings.^15^ However, more context-specific modelling is needed to evaluate the cost-effectiveness of such strategies in South Africa. The recent adoption of the WHO-endorsed single-dose HPV vaccine schedule in South Africa,^16^ along with the availability of more affordable vaccines, offers promising opportunities to enhance economic feasibility.

## Recommendations

To strengthen HPV prevention and protect neonatal health, the following actions are recommended:

- Adopt a gender-neutral HPV vaccination policy targeting all eligible adolescents, regardless of sex or gender, within the school-based health programme
- Raise awareness among healthcare providers, educators and parents about the risks of vertical transmission and the benefits of vaccinating boys
- Expand research into the vertical and perinatal effects of HPV, including longitudinal studies that track neonatal outcomes and long-term vaccine impact
- Conduct context-specific modelling studies that incorporate vertical transmission and broader HPV-related complications in cost-effectiveness analyses
- Ensure equitable access to the HPV vaccine across diverse socioeconomic and geographic populations to prevent the widening of existing health disparities.

## Conclusion

Human papillomavirus is not solely a women’s health issue or a disease that only emerges in adulthood. Its consequences can appear much earlier, including during pregnancy and at birth. While current evidence linking neonatal complications to parental HPV infection is limited, it raises important questions about parent-to-child risks. Implementing a gender-neutral HPV vaccination strategy for adolescents in South Africa presents a proactive and ethically sound opportunity to prevent HPV-related complications across the lifespan. Protecting the health of neonates begins with protecting their parents through inclusive, evidence-informed and socially just public health strategies.
